# Use of an Artificial Miniaturized Enzyme in Hydrogen Peroxide Detection by Chemiluminescence

**DOI:** 10.3390/s20133793

**Published:** 2020-07-06

**Authors:** Gerardo Zambrano, Flavia Nastri, Vincenzo Pavone, Angela Lombardi, Marco Chino

**Affiliations:** Department of Chemical Sciences, University of Naples “Federico II”. Via Cintia, 80126 Napoli, Italy; gerardo.zambrano@unina.it (G.Z.); flavia.nastri@unina.it (F.N.); vincenzo.pavone@unina.it (V.P.); angela.lombardi@unina.it (A.L.)

**Keywords:** luminescence, hydrogen peroxide, heme proteins, artificial metalloenzymes, luminol

## Abstract

Advanced oxidation processes represent a viable alternative in water reclamation for potable reuse. Sensing methods of hydrogen peroxide are, therefore, needed to test both process progress and final quality of the produced water. Several bio-based assays have been developed so far, mainly relying on peroxidase enzymes, which have the advantage of being fast, efficient, reusable, and environmentally safe. However, their production/purification and, most of all, batch-to-batch consistency may inherently prevent their standardization. Here, we provide evidence that a synthetic de novo miniaturized designed heme-enzyme, namely Mimochrome VI*a, can be proficiently used in hydrogen peroxide assays. Furthermore, a fast and automated assay has been developed by using a lab-bench microplate reader. Under the best working conditions, the assay showed a linear response in the 10.0–120 μM range, together with a second linearity range between 120 and 500 μM for higher hydrogen peroxide concentrations. The detection limit was 4.6 μM and quantitation limits for the two datasets were 15.5 and 186 μM, respectively. In perspective, Mimochrome VI*a could be used as an active biological sensing unit in different sensor configurations.

## 1. Introduction

According to the United Nations (UN) and World Health Organization (WHO) reports about the consequences of climate change, extreme weather events and variable climates are affecting food and water supplies [1,2]. Moreover, free access to drinking water is considered a fundamental and universal human right [3]. In this context, water reclamation for potable reuse is nowadays considered a necessary approach to face near-future water scarcity. Among the chemical, physical, and biological treatments to which reclaimed water must be subjected, advanced oxidation processes (AOPs), coupling either UV irradiation or ozonation in the presence of hydrogen peroxide, are effective both in microbial sterilization and organic pollutant oxidative degradation [4,5,6]. Hydrogen peroxide determination is, therefore, crucial in: (i) Assessing the undesired residual peroxide concentration of the final treated water; (ii) monitoring, hopefully on a real-time basis, the process performance; (iii) tuning the reagent amount in order to get the best results in terms of its ecological and economic costs.

**Scheme 1:**
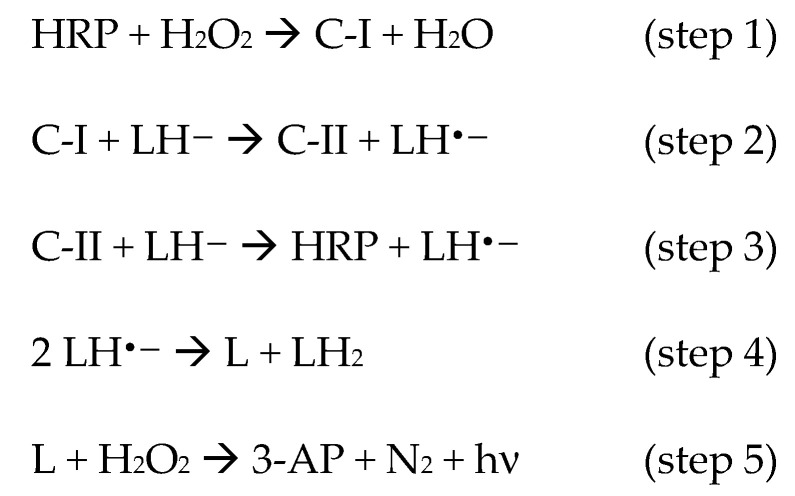
Mechanism of horseradish peroxidase-catalyzed luminol oxidation by hydrogen peroxide, and subsequent luminescence.

The determination of hydrogen peroxide is a historically fervent research field, as its concentration in solution is directly or indirectly related to the activity of several enzymes [7,8]. Many bio-based assays have been developed with potential food, clinical, and biotechnological applications [9,10,11,12,13,14,15,16,17,18,19,20,21,22,23,24,25,26,27,28,29,30,31,32,33]. Different techniques may be coupled to these assays, such as spectrophotometry [9], fluorimetry [10,11], electrochemistry [12,13,14,15,16,17], and chemiluminescence (CL) [18,19,20,21,22,23,24,25,26,27,28,29,30,31,32,33], giving rise to a wide spectrum of sensitivities and variable ranges of detection. CL offers several advantages, such as the use of very sensitive and miniaturized detectors, from lab bench PMT detectors to smartphone CCD cameras, and the almost complete absence of background signal [8]. To this aim, luminol has been widely preferred over other CL reagents thanks to its high quantum yield, enabling the detection of trace amounts of materials [7,23,24,25,26,27,28,34]. Upon oxidation, luminol (LH_2_) develops blue light with an emission maximum at 425 nm. Several oxidation conditions have been reported in the literature, involving either catalyzed activation of hydrogen peroxide in alkaline medium, or *in situ* formation of singlet dioxygen species (^1^O_2_) [26,35,36,37,38]. Biomolecule-based activation of hydrogen peroxide has several advantages over other methods, it is generally highly selective and efficient, the catalyst can be recycled, and the use of toxic reagents is avoided [22,23,24,25]. Horseradish peroxidase (HRP) catalyzes hydrogen peroxide activation and further oxidation of luminol, thus generating a moderately high and durable luminescence signal (LS). The mechanism of luminol oxidation by HRP has been studied in detail and involves several steps before light emission [37,39,40,41,42,43,44]. First, hydrogen peroxide induces the formation of the high oxidation state compound I (C-I) intermediate (Scheme 1; step 1), which, in turn, oxidizes two equivalents of luminol, in the deprotonated form LH^—^ under alkaline conditions, through the formation of the compound II (C-II) intermediate (Scheme 1; steps 2–3). Subsequently, rapid dismutation of the early produced radical species yields the fully oxidized diazaquinone (L; Scheme 1; step 4). Finally, an uncatalyzed coupling between a second equivalent of H_2_O_2_ and L takes place in the rate-limiting step, which gives rise to dinitrogen release and formation of 3-aminophtalate (3-AP) in an excited triplet state (Scheme 1; step 5). Intersystem crossing then leads to the decay toward the emissive singlet state.

Suitability of the HRP/luminol reaction system in the determination of hydrogen peroxide has been recognized many years ago [18]. Both batch and flow assays have been developed, even though only a limited response, in terms of linearity range and limit of detection (LOD), could be achieved [9,21,22,23,24,25]. A substantial upgrade of the sensing capacity was accomplished both by enzyme immobilization onto different matrices and by LS enhancers [9,22,23,24,25,45,46]. LS could be modulated by the presence of different organic molecules (generally aromatic), detergents, and metal ions, both in the enhancement and in the suppression of the emitted light, allowing the determination of several analytes [23,24,25]. Finally, bi-enzymatic sensors could be developed by coupling HRP/luminol LS either to H_2_O_2_-dependent enzymes, as glucose oxidase (GOx) enzyme for glucose determination, or to highly selective antibodies for antigen detection [34,47].

**Figure 1:**
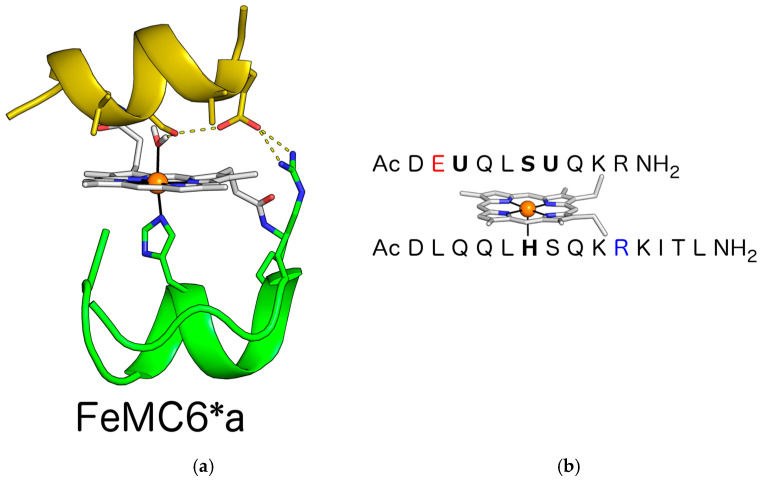
(**a**) FeMC6*a designed model. Key functional residues and the deuteroporphyrin IX are highlighted as sticks. Iron is represented as an orange sphere. Aib residues face the porphyrin ring at the distal site. The designed inter-chain ion pair interaction is depicted, together with the hydrogen bond network presumably involved in hydrogen peroxide activation. (**b**) FeMC6*a peptide sequences. Proximal His and distal axial residues are indicated in bold. The Glu and Arg residues involved in the inter-chain ion pair interaction are depicted in red and blue, respectively.

Artificial metalloenzymes (ArMs) have shown their versatility in modeling natural functions as well as in engineering catalytic sites able to perform uncommon or non-natural substrate conversion [48,49,50,51,52]. In this respect, we developed different metal-binding sites in the context of either four-helix bundle [53,54] or peptide-porphyrin conjugates [55,56,57]. In the former scaffold, we afforded the design of non-heme di-iron oxidases and oxygenases, as well as the stabilization of an unprecedented tetra-zinc metal cofactor [58,59,60,61]. In the latter, we miniaturized and then reengineered the heme-binding site of globins, obtaining a tailor-made set of catalysts named Mimochromes (MC) [62,63,64,65,66,67,68]. The MC scaffold recovers many natural hemeprotein prerogatives, by mimicking the first and second coordination sphere interactions around the metal cofactor. MCs comprise two small peptide chains covalently linked to deuteroporphyrin and arranged to fold into a helix–heme–helix sandwich (Figure 1a). Over the years, we rationally evolved the construct from a coordinatively saturated non-catalytic complex to penta-coordinated catalytic analogs [62,63,64]. An iterative process of design and redesign allowed us to engineer and optimize the peroxidase activity into an MC scaffold, thus affording MC6*a (Figure 1a) [65]. MC6*a comprises: A tetradecapeptide (TD) chain bearing a His residue acting as the heme axial proximal ligand; a decapeptide (D) chain, lacking the metal coordinating residue, thus resembling the distal site of heme-peroxidase for accommodating hydrogen peroxide. The helix–heme–helix sandwich structure is stabilized by an *inter*-chain salt bridge between the Glu2 residue in the (D) chain and the Arg10 residue in the (TD) chain. Further, two 2-aminoisobutyric acid (Aib), at positions 3 and 7 of the (D) chains, stabilize the helical conformation and drive the peptide chain to face the metalloporphyrin ring, thanks to hydrophobic interactions (Figure 1b). 

FeMC6*a overcomes the catalytic activity of HRP in ABTS oxidation [65] and in thioanisole oxygenation [66]. This minimal scaffold showed higher resistance against oxidative damage, thus highlighting the protective role exerted by the peptide matrix on the metal cofactor, and it was able to tune the reactivity of the metalloporphyrin. Indeed, MC6*a manganese complexes were found to be a competent catalyst in peroxygenase activities [66], whereas the cobalt derivative was active in hydrogen evolution catalysis [67,68].

Substituting natural enzymes with properly designed metalloenzymes would be valuable, as fine-tuning the enzyme performances by design should open the way to the construction of tailor made catalysts. The interesting results obtained on the MC6*a artificial enzyme prompted us to exploit its practical application. To this end, we have recently reported the feasibility of FeMC6*a as a clickable artificial peroxidase, which easily reacts with azide-functionalized molecules and/or nanomaterials to afford functional bioconjugates [69]. Further, the ability of FeMC6*a to overcome HRP catalytic behavior suggested its application as a substitute of natural enzymes in biosensor technology. 

In this study, we describe the FeMC6*a proficiency in catalyzing the luminol oxidation and further light emission. First, we compared the activities of FeMC6*a to commercial HRP. Then, we developed a batch assay for H_2_O_2_ determination, which can be performed with a simple microplate reader, by screening the best reaction conditions in terms of pH, enzyme, and luminol concentrations. Given the complex kinetic interplay of the reaction steps, the outcome of these experiments is not trivial, and they could be interpreted in terms of the mechanism reported for the natural peroxidase. Finally, we showed that FeMC6*a induces H_2_O_2_-dependent LS, which is linear over a wider range of concentrations, compared to HRP, and we tested this in the simulated AOP of a reducing aromatic compound. 

## 2. Materials and Methods

### 2.1. Reagents

All reagents have been purchased from Sigma-Merck, if not differently specified. HRP Type VI-A (batch SLBH1737V) lyophilized powder was at > 950 U/mg (using ABTS) and directly renatured under the assayed buffer conditions (ε(403) = 100 mM^−1^ × cm^−1^). Hydrogen peroxide and FeMC6*a stock solutions were prepared using 39.4 M^−1^ × cm^−1^ (at 240 nm) and 117 mM^−1^ × cm^−1^ (at 387 nm) as the molar absorptivities, respectively. 

### 2.2. Enzyme Synthesis and Purification

MC6*a free base has been synthesized as previously reported [65]. Iron insertion has been accomplished by a slightly modified acetate method, as reported elsewhere [63]. FeMC6*a purity has been ascertained by LC–MS analysis (LC-20 Prominence coupled to an ESI IT-TOF high-resolution mass spectrometer, Shimadzu Corporation, Japan) to be higher than 95%.

### 2.3. Luminescence Standard Assay

The standard assay was prepared in a 135 μL final volume reaction mixture containing 0.3 μM FeMC6*a and 0.100 mM luminol. After 2 min of incubation at 25 °C, aliquots of H_2_O_2_ stock solutions (15 μL) were added to the assay solution. Chemiluminescence measurements were carried out in a TECAN Spark plate reader (Tecan Trading AG, Switzerland) using a 96-well plate (Greiner PS Microplate, 96 Well, solid F-bottom (flat), chimney well with black sides). The rate of luminol oxidation was determined by monitoring the increase in luminescence at 425 nm. Molar absorptivity of 7630 M^−1^ × cm^−1^ at 347 nm was used for luminol standardization [40]. All measures were carried out at least three times, arbitrary units (a.u.) have been reported by dividing the instrumental response (counts per second) by 10^6^, and the data were analyzed by the software OriginPro 8 (Copyright 1991–2007 OriginLab Corporation, Northampton, MA, USA).

### 2.4. Steady-State Luminescence Kinetics

Steady-state luminescence kinetic traces were recorded on a FluoroMax 4 (Horiba, Minami-ku Kyoto, Japan) instrument in the absence of any excitation (5 nm emission slit width). Temperature control was kept by an external Peltier unit. A reduced volume four-windowed cell (10/3.3 mm path lengths) was used and agitation was granted by a stirring bar. The assays were carried out in a dark room by injecting 10 μL of H_2_O_2_ to a final concentration of 3 mM with a Hamilton syringe into a 2 mL solution of 0.1 M Tris/HCl pH 8.5 buffer containing 0.5 mM luminol and 2 nM catalyst. 

### 2.5. Simulated Advanced Oxidation Process of Thioanisole

Two four-windowed quartz cells (10 mm path lengths) were filled with 2.5 mL of either water or 81 μM thioanisole. H_2_O_2_ was then added to both cuvettes to a final concentration of 8.1 mM. Then, the samples were exposed to a UV lamp photopolymerization apparatus (type Zp lamp UV-A/B/C at 500 W, HELIOS ITALQUARTZ, Milan, Italy) for two hours. Further, 200 μL aliquots were collected at time zero and after each hour and stored at 4 °C in dark. Thereafter, all the collected aliquots were processed to the luminol assay according to the procedure reported in Section 2.3, with the exception that aliquots taken at time zero were primarily diluted 1:4 with water.

## 3. Results and Discussion

### 3.1. Assessment of the Artificial Peroxidase Proficiency in Luminol Oxidation

**Figure 2:**
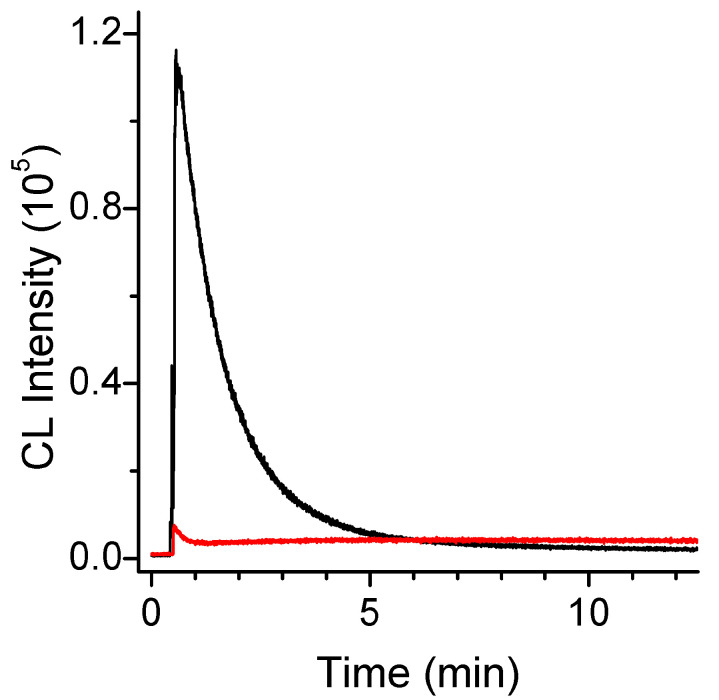
Chemiluminescence kinetic trace of luminol/H_2_O_2_ (0.5 mM/3 mM) system when 2 nM FeMC6*a (black trace) or HRP (red trace) is used as a catalyst (100 mM Tris buffer pH 8.5).

In order to test whether FeMC6*a could proficiently catalyze luminol oxidation by activation of hydrogen peroxide, we performed a preliminary steady-state kinetic study of LS production by FeMC6*a, and compared it to that obtained by HRP in the absence of any enhancer. When 3 mM hydrogen peroxide was added to a solution containing 0.5 mM of luminol and 2 nM of FeMC6*a, a very intense LS at 425 nm was developed that lasted more than 10 min, corresponding to the relaxation time of the 3-AP [35] (Figure 2). 

A very brilliant LS peak approached its maximum after approximately 1 min and then rapidly quenched, following a kinetic trace that has been previously observed in the literature when a very high concentration of peroxidase was adopted [19,37,44], or when luminol was in high excess with respect to hydrogen peroxide [41,42,43,44,45]. Instead, in the presence of HRP as a catalyst, only a modest LS was observed in the first 600 s, under these conditions, as previously reported for this catalytic system in the absence of enhancers [46].

**Figure 3:**
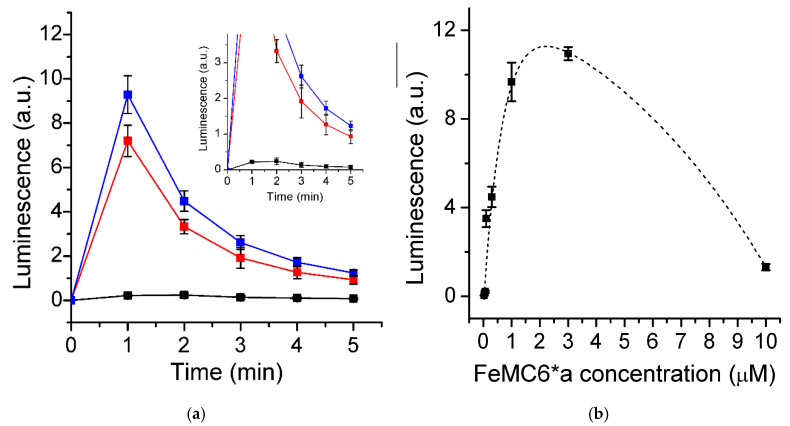
(**a**) Luminescence signal (LS) kinetic traces as registered at different FeMC6*a concentrations (0.07 μM, black; 0.1 μM, red; 0.3 μM blue). Insert shows the enlargement of the LS traces. Each point is the mean of three measurements, with error bars representing the standard deviations. (**b**) FeMC6*a concentration dependence of chemiluminescence as measured at 2 min after H_2_O_2_ addition in the plate reader (0.5 mM H_2_O_2_, 10 mM luminol, 100 mM Tris buffer pH 8.5). Dashed line corresponds to the best biexponential fit of the experimental data.

These encouraging results prompted us to elaborate a CL-based hydrogen peroxide assay that could be easily monitored by a bench microplate reader. Given the different sensitivity of these kind of instrument, and the different geometry of the experimental setup, we first tested the best conditions in terms of sampling time and optimal FeMC6*a concentration under very harsh conditions of H_2_O_2_/luminol (0.5/10 mM). Kinetic traces of LS as taken by the plate reader under different FeMC6*a concentrations show a strong signal after 1 min, as previously shown for the steady-state kinetic trace. However, we preferred to take our measurements at 2 min to decrease the extent of variability in the LS value (Figure 3a). The signal intensity (herein expressed in a.u., see Materials and Methods), read after 2 min, increased almost exponentially with FeMC6*a concentration up to 1 μM, slowly decaying thereafter to very low values at 10 μM concentration (Figure 3b). 

The observed trend has been previously documented, and it is expected when considering that at very high amounts of catalyst, hydrogen peroxide is rapidly and entirely consumed to produce the luminol radical, and no H_2_O_2_ is then available to react with the diazaquinone (formed upon radical dismutation) to give the 3-AP (Scheme 1) [41,42,43,44,45].

Based on the FeMC6*a concentration dependence of LS (see Figure 3b), we selected 0.3 μM as the FeMC6*a concentration to be used for the optimization of other experimental conditions. This concentration appeared to us as a good compromise to obtain a strong signal without detector saturation. 

### 3.2. Effect of pH and Luminol Concentration

**Figure 4:**
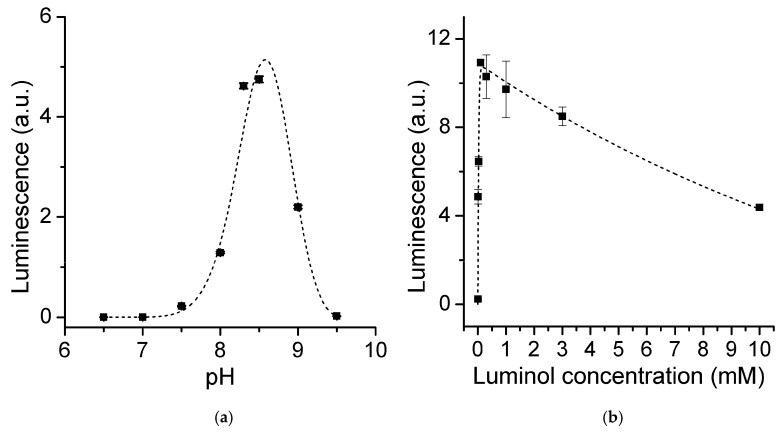
(**a**) Effect of the pH on LS, as measured at 2 min after hydrogen peroxide addition. (0.3 μM FeMC6*a, 0.5 mM H_2_O_2_, 10 mM luminol, 100 mM Tris buffer). Dashed line corresponds to the best Gaussian fit of the experimental data. (**b**) Effect of luminol concentration on LS (0.3 μM FeMC6*a, 0.5 mM H_2_O_2_, 100 mM Tris buffer pH 8.5). Dashed line corresponds to the best biexponential fit of the experimental data.

Our preliminary results prompted us in screening for the best conditions for the peroxide-dependent CL assay. The optimal pH for the peroxidase activity of FeMC6*a was previously found to be 6.5 [65] in other oxidation reactions; however, the LS efficiency of luminol was maximum around pH 10 [18,22,25] where a luminol deprotonated form is abundant. The maximum LS is therefore expected in the range of 6.5–9.5. The LS was measured at 2 min after the hydrogen peroxide addition at various pH in the range of 6.5–9.5. We found optimal LS at pH of 8.5 (Figure 4a), quite similar to that reported for HRP [18,22,25]. MC6*a and HRP catalytic activities are practically indistinguishable in terms of pH dependence, making the artificial metalloenzyme a viable alternative to the natural enzyme in this specific application.

Given that luminol is involved in several reaction steps, luminol concentration is an important variable to optimize. In this respect, we decreased the luminol concentration from 10 mM down to 3 μM under the optimized FeMC6*a concentration and pH and kept the H_2_O_2_ concentration constant at 0.5 mM. The observed LS vertically increased with the increase in luminol initial concentration and reached its maximum at 0.1 mM (Figure 4b).

**Figure 5:**
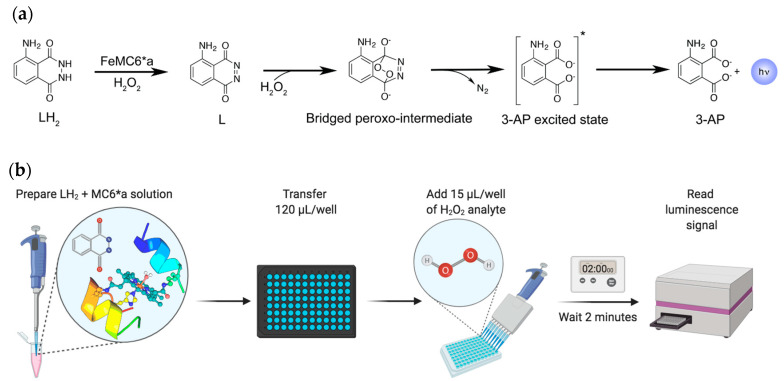
(**a**) Proposed reaction scheme of luminol oxidation catalyzed by FeMC6*a. (**b**) Step-by-step hydrogen peroxide assay developed in this work.

The further increase in luminol concentration caused an exponential drop in the signal, approaching 4 a.u. at luminol concentrations higher than 10 mM. As previously discussed, the multi-step mechanism leading to CL involves the consumption of two equivalents of H_2_O_2_ in two steps as shown in Scheme 1 and in Figure 5a. 

Therefore, one would expect that LS would be maximized when the hydrogen peroxide to luminol ratio is 2:1. However, luminol radical formation (LH^•–^) is generally considered faster than the subsequent diazaquinone (L) oxidation [42]. Given that LS depends on the latter (Figure 5a), a higher amount of H_2_O_2_ is actually needed to maximize the signal, for two reasons: (i) To increase the rate for the uncatalyzed second-order reaction between H_2_O_2_ and L; (ii) to prevent total consumption of H_2_O_2_ in the first oxidation step. Our results indeed show that maximum LS is reached at 0.1 mM luminol, when 0.5 mM hydrogen peroxide was used (5:1 hydrogen peroxide:luminol ratio). Therefore, we adopted this luminol concentration for quantitative hydrogen peroxide assay development. 

### 3.3. Hydrogen Peroxide Determination

**Figure 6:**
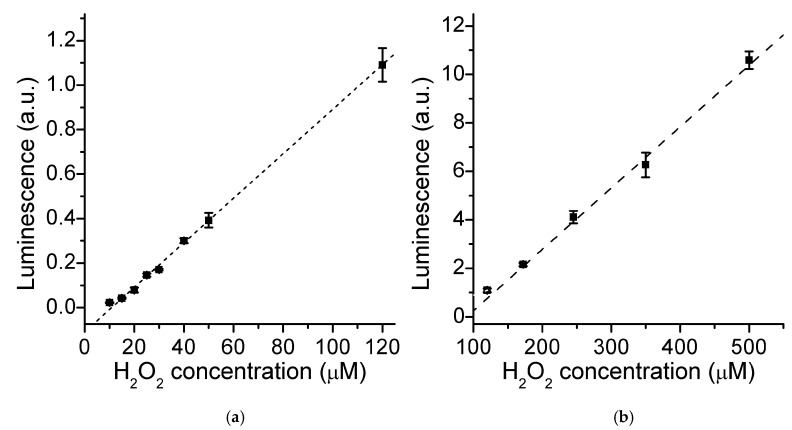
(**a**) LS response to H_2_O_2_ concentration, as measured at 2 min after hydrogen peroxide addition in the 10.0–120 μM range. (0.3 μM FeMC6*a, 0.1 mM luminol, 100 mM Tris buffer pH 8.5) (**b**) LS observed at different H_2_O_2_ concentrations in the 120–500 μM range. Short- and long-dashed lines correspond to the best linear fits of the two datasets, respectively.

Under the optimized conditions (0.3 μM FeMC6*a, 0.1 mM luminol, pH 8.5, 2 min), the LS response to H_2_O_2_ concentration was followed according to the developed procedure (Figure 5b). A bimodal linear trend was found (Figure 6). LS increased slowly and linearly at H_2_O_2_ concentrations lower than 120 μM, whilst LS increased more linearly and steeply at higher concentrations. Nonetheless, the observed lag-phase gave us the opportunity to extrapolate a linear interval of response in the range of 10.0–120 μM (Figure 6a). 

The sensitivity (slope of the linear fit) was found to be 10.1 10^−3^ a.u./μM, the agreement was very good, and a detection limit (LOD) of 4.6 μM and quantitation limit (LOQ) of 15.5 μM were calculated from the y-residuals (R^2^ = 0.9997, n = 24). At peroxide concentrations higher than 120 μM (Figure 6b), more intense signals are observed, and a linear fit of the data was obtained (R^2^ = 0.991, n = 15), with the LOD far below the observed linearity range (55.8 μM) and the LOQ being 186 μM (0.12–0.50 mM; 25.0 10^−3^ a.u./μM).

By contrast, HRP showed a typical Michaelis–Menten dependence of the LS response toward H_2_O_2_ concentration [41,42]. Nonetheless, a nonlinear response against H_2_O_2_ concentration in the pre-saturation phase was previously reported by many authors in the case of HRP and lactoperoxidase in batch assays, when a high concentration of the catalyst was used [22,42,44]. Although a more detailed study of the kinetic mechanism of FeMC6*a is required, on the basis of the HRP mechanism proposed by Arnold and co-workers [42], it appeared reasonable to us to hypothesize that: (i) When the [H_2_O_2_]/[luminol] ratio is lower than 1, H_2_O_2_ activation (Scheme 1; step 1) and diazaquinone oxidation are both rate-limiting, affecting LS changes; (ii) when the ratio is higher than 1, diazaquinone is rapidly formed (within 2 min) and LS rapidly increases with exceeding H_2_O_2_.

Interestingly, the increased CL efficiency of FeMC6*a enables H_2_O_2_ detection in the μM range, as compared to the mM range when HRP is used as a catalyst [22]. In order to circumvent such limitation, HRP has been either coupled to an CL enhancer [23,24], or immobilized [25], increasing the signal-to-noise ratio and response linearity. Given the observed analogies between FeMC6*a and HRP, we expect that such approaches could lead to further enhancement of FeMC6*a performances in luminol oxidation.

### 3.4. Sample Analysis and Organic Contaminant Interference Study

**Figure 7:**
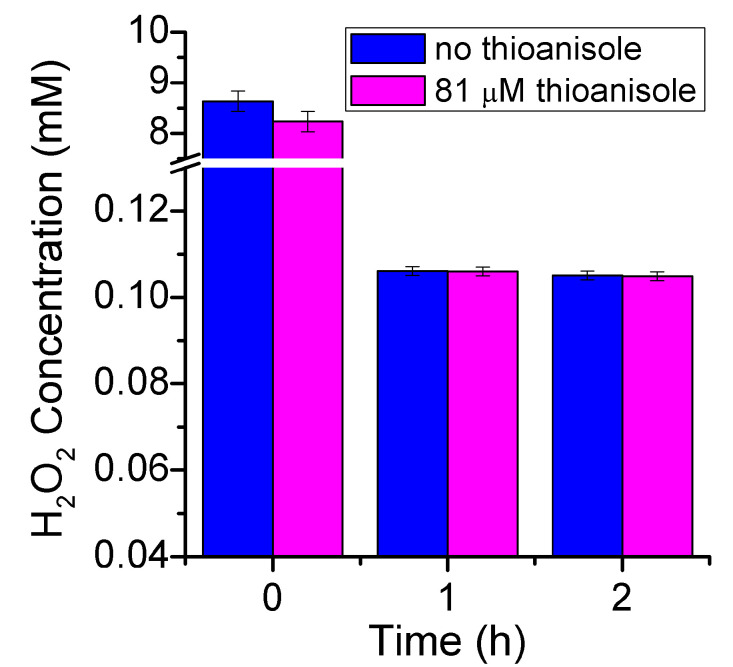
Advanced oxidation processes (AOPs) as followed by the FeMC6*a/luminol assay. Blue (without thioanisole) and magenta (81 μM thioanisole) columns report the evaluated H_2_O_2_ concentration before and after 1 and 2 h of UV-light irradiation. Thin bars report the statistical error for data regression.

The utility of the assay developed here was checked by simulating a real-case scenario of AOP. Two samples were prepared either with or without 10 ppm thioanisole as organic contaminant, in which H_2_O_2_ was added to a final concentration of 8.1 mM (1:100 thioanisole:H_2_O_2_ ratio). Both samples were then exposed to UV irradiation for two hours to induce hydroxyl radical (OH^•^) formation and oxidation of the organic matter. Subsequently, various aliquots were taken at different times to measure the H_2_O_2_ content by the proposed FeMC6*a/luminol assay (Figure 7). 

The assay was able to recover the starting concentration of hydrogen peroxide, being only slightly overestimated in the sample without thioanisole. In fact, the measured concentrations were 8.2 ± 0.2 and 8.6 ± 0.2 mM for samples with and without thioanisole, respectively (106% and 101% recovery). Virtually no difference could be observed within the experimental error between the two samples after one and two hours. Very strong consumption of hydrogen peroxide was observed in the first hour (106 ± 1 μM), while only a minimal decrease in H_2_O_2_ concentration was observed after the second hour of UV-light exposure (105 ± 1 μM). 

## 4. Conclusions

In the present work, the artificial enzyme, FeMC6*a, was applied in the development of a synthetic peroxidase-based batch-assay for H_2_O_2_ determination. Notably, FeMC6*a proved itself as an extremely proficient catalyst in luminol oxidation, despite its miniaturized scaffold. In comparison to HRP-based CL sensors for H_2_O_2_, FeMC6*a-catalyzed oxidation of luminol and further light emission detection proceed within a short time range (~2 min) without the need of enhancers. An efficient detection of H_2_O_2_ was observed using low concentrations of enzyme (0.3 µM; 1.05 mg/L) with good linearity both in the micromolar (R^2^ = 0.9997, *n* = 24) and in the low millimolar (R^2^ = 0.991, *n* = 15) ranges. LOD and LOQ were satisfactory, being as low as 4.6 and 15.5 μM, respectively. Moreover, the synthetic origin of FeMC6*a provides batch-to-batch consistency, paving the way to method standardization and reduced batch-to-batch discrepancies. 

This work has demonstrated that FeMC6*a can be adopted in luminescence-based sensors. Moreover, the results obtained from a real-case scenario not only show that the reported assay is able to satisfactorily recover the starting H_2_O_2_ concentration but also that H_2_O_2_ concentration could be actually monitored during an AOP test. We found that H_2_O_2_ was not completely consumed within two hours of UV-light irradiation, thus demonstrating the suitability of the FeMC6*a/luminol assay to assess undesired residual hydrogen peroxide content. Moreover, the presence of 10 ppm of a well-known reducing aromatic compound did not influence the assay outcome, demonstrating the usability of FeMC6*a for this kind of application in hydrogen peroxide sensing. Future efforts will be devoted to enhance the performances of FeMC6*a in luminol-based H_2_O_2_ determination either by using enhancers or immobilization onto solid supports [16,19,23,25,30,31,64], and to the construction of a standalone sensor device. Process control and quality assessment of reclaimed water may be further improved by developing efficient multi-purpose devices [4,28]. In particular, different oxidants, other than hydrogen peroxide, such as chlorite and chlorine dioxide [70], may be revealed through the methodology developed here, and their consumption rate may be eventually coupled to the presence of several aromatic and organophosphate pollutants [71,72,73].
